# In Vitro Whole-Genome Analysis Identifies a Susceptibility Locus for HIV-1

**DOI:** 10.1371/journal.pbio.0060032

**Published:** 2008-02-19

**Authors:** Corinne Loeuillet, Samuel Deutsch, Angela Ciuffi, Daniel Robyr, Patrick Taffé, Miguel Muñoz, Jacques S Beckmann, Stylianos E Antonarakis, Amalio Telenti

**Affiliations:** 1 Institute of Microbiology, University Hospital, University of Lausanne, Lausanne, Switzerland; 2 Department of Genetic Medicine and Development, University of Geneva Medical School and University Hospital of Geneva, Geneva, Switzerland; 3 Swiss HIV Cohort Study, Lausanne, Switzerland; 4 Department of Medical Genetics, University Hospital and University of Lausanne, Lausanne, Switzerland; University of Alabama, United States of America

## Abstract

Advances in large-scale analysis of human genomic variability provide unprecedented opportunities to study the genetic basis of susceptibility to infectious agents. We report here the use of an in vitro system for the identification of a locus on HSA8q24.3 associated with cellular susceptibility to HIV-1. This locus was mapped through quantitative linkage analysis using cell lines from multigeneration families, validated in vitro, and followed up by two independent association studies in HIV-positive individuals. Single nucleotide polymorphism rs2572886, which is associated with cellular susceptibility to HIV-1 in lymphoblastoid B cells and in primary T cells, was also associated with accelerated disease progression in one of two cohorts of HIV-1–infected patients. Biological analysis suggests a role of the rs2572886 region in the regulation of the LY6 family of glycosyl-phosphatidyl-inositol (GPI)–anchored proteins. Genetic analysis of in vitro cellular phenotypes provides an attractive approach for the discovery of susceptibility loci to infectious agents.

## Author Summary

Individuals differ in their susceptibility to the HIV-1 virus, and the determinants of susceptibility are encoded in the human genome. Genetic variants influencing this trait have been identified by investigating candidate genes thought likely to be involved in HIV-1 pathogenesis or by whole-genome association studies, which type more than 500,000 genetic variants per individual (genome-wide association studies) to see which ones associate with susceptibility. We have addressed the issue of identification of new genetic variants influencing susceptibility to HIV-1 by a novel strategy based on the in vitro infection of cells. For this, immortalized B lymphocytes from 15 families (198 cell lines) were infected by a HIV-based vector. Differences in cellular susceptibility to infection—a genetic trait—could be mapped to a precise region on Chromosome 8, suggesting a role of the LY6 family of GPI-anchored proteins in HIV-1 infection. Genetic analysis of in vitro standardized cellular phenotypes provides a new approach to the discovery of the basis of genetic susceptibility to infectious agents.

## Abstract 3

Susceptibility to HIV is, to a significant extent, determined by the human genetic background. The authors infected immortalized B lymphocytes from 15 large-pedigree families and mapped a locus in Chromosome 8 associated with HIV susceptibility.

## Introduction

Some individuals do not become infected to the HIV-1 virus despite repeated exposures, and among those that do, there is marked variation in the clinical course and progression to AIDS [1]. Although a number of host genetic determinants of susceptibility to HIV-1 have been identified through the analysis of candidate genes—most notably CCR5 Δ32 and HLA alleles—only a fraction of the observed phenotypic variation can be explained by variation at these loci [2,3]. Thus, there is a considerable interest in applying unbiased methods such as whole-genome analysis for the identification of novel susceptibility loci to human pathogens [1]. This hunt is, however, plagued by numerous confounding factors such as the lack of ascertainment of informative patient cohorts and difficulties to control for the variability of the infectious agent. Whole-genome mapping for viral susceptibility has been reported in mice for the murine adenovirus type 1 [4], and in mosquitoes for the dengue-2 virus [5]. Recently, the first genomewide association analysis for determinants of host control of HIV-1 in humans has been completed [6].

Whole-genome scans can also be performed through the analysis of family data using linkage analysis, an approach widely used to map monogenic disorders [7,8]. The need for family-based data has limited the use of this approach in the HIV-1 field because of the rarity, beyond instances of vertical transmission, of multi-case family infections. Studies of host genetic susceptibility to HIV-1 are also confounded by differences in virulence of the infecting viral strain [1].

To circumvent these limitations, we established an in vitro system to address the genetic control of cellular susceptibility to HIV-1 using cell lines from multi-generation families [9,10]. We used families from the Centre d'Etude du Polymorphisme Humain (CEPH) resource (up to four grandparents and an average of eight children per family), consisting of Epstein-Barr virus (EBV)–immortalized lymphoblastoid B cell lines (LCL). CEPH LCLs have been extensively genotyped, and the data are publicly available (http://snpdata.cshl.edu/population_studies/linkage_maps//). CEPH LCLs were previously used to identify genomic loci influencing sodium–lithium counter transport [11], natural variation in gene expression [12–15], transcriptional response to ionizing radiation [16], susceptibility to chemotherapy [17], and the relative impact of nucleotide and copy number variation on gene expression [18,19]. We hypothesized that CEPH LCLs could allow genome-wide investigation of interindividual variation of cellular susceptibility to infection with an isogenic virus, under standardized conditions and a controlled environment.

## Results/Discussion

**Figure 1:**
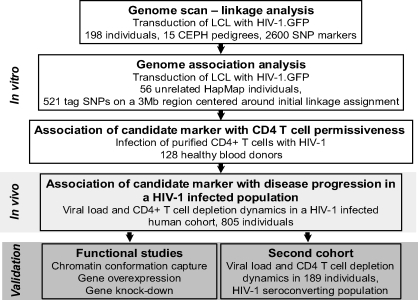
Study Flowchart LCL, lymphoblastoid B cell lines; HIV.GFP, vesicular stomatitis virus G protein-pseudotyped HIV-1-based vector; CEPH, Centre d'Etude du Polymorphisme Humain; SNP, single nucleotide polymorphism.

We designed the study to progress through consecutive steps including: (i) the identification by linkage and association of candidate markers associated with in vitro cellular susceptibility to an HIV-1-based vector*,* (ii) in vivo validation of candidate polymorphisms in humans infected with HIV, (iii) investigation of potential biological mechanisms (Figure 1).

Since B cells are not a natural target of HIV-1, we established the conditions for efficient transduction of lymphoblasts with a VSV.G (vesicular stomatitis virus G protein)-pseudotyped HIV-1–based vector (HIV.GFP). We first assessed to what extent immortalized B cells reflect the behavior of CD4^+^ T cells by transducing purified CD4^+^ T cells and EBV-immortalized B cells, from the same 11 Caucasian healthy blood donors, with the same HIV.GFP. We used the green fluorescent protein (GFP) transgene expression as a reporter for permissiveness to lentiviral infection. We observed a significant correlation (Pearson *r* ^2^ = 0.56, *p* = 0.007) between the level of transduction of CD4^+^ T cells and B lymphoblastoid cells for the same individuals (Figure S1). Thus, we hypothesized that transduction of B cells can capture a significant proportion of interindividual variation of post-entry events in the HIV-1 life cycle (reverse transcription, integration, transcription, and translation). Additional validation of the assay established the intra- and interday reproducibility of the transduction phenotype in CEPH LCLs, and ruled out an influence of potential confounders such as EBV copies per cell and the level of expression of the EBV-transforming protein LMP1 (unpublished data).

To determine whether variation in cellular susceptibility to the HIV.GFP virus has a genetic component we estimated heritability (*h* ^2^, i.e., the proportion of variance attributable to additive genetic factors) in five CEPH pedigrees (76 individuals). In parallel, we also scored eight additional traits unrelated to HIV susceptibility (EBV copy number, EBV LMP1 oncogene CD11a, CD19, CD21, CD23, CD39, CD54). We observed a significant heritability of the HIV susceptibility trait (*h* ^2^ = 0.54, *p* = 1.6 ×10^−6^), as well as for most of the other traits, with *h* ^2^ values in the same range as those reported for gene expression variation traits [20] (Figure S2).

In view of these significant heritability results, we extended the analyses to 15 CEPH pedigrees (198 individuals) for the lentiviral cellular permissiveness trait (Figure S2), and we selected the expression of the endogenous cell surface marker CD39 (EBV receptor), and of the EBV-encoded LMP1 protein as additional study phenotypes.

**Figure 2:**
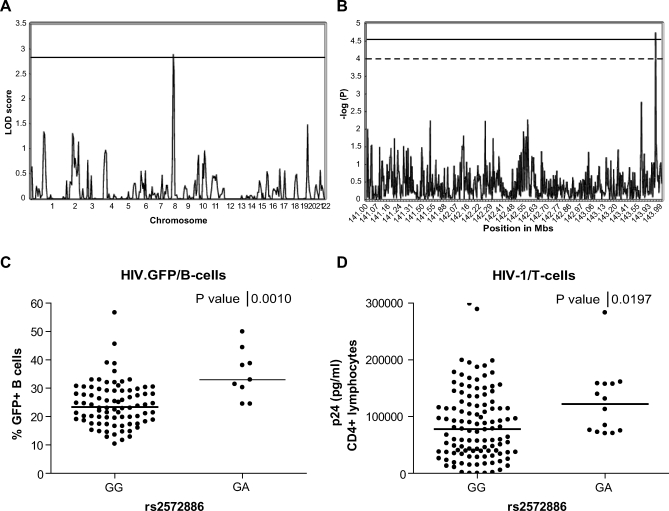
Genome Scan and Fine Mapping of an HIV-1 Susceptibility Locus, and In Vitro Validation of the Candidate SNP Marker (A) Linkage analysis with 15 families and 2,600 markers identifies a quantitative trait locus (QTL) on HSA8q24.3. The highest multipoint LOD score of 2.89, *p* = 1.3 × 10^−04^ (marker rs1398296) was significant at genome-wide level as determined by permutation analysis (95% significance threshold of 2.83, dotted line). (B) Association analysis in 56 unrelated individuals, using 521 SNPs from the HapMap project centered 3 Mb around the initial QTL, identifies marker rs2572886 (*p* = 1.8 × 10^−5^). The association remained significant after correction for multiple testing by Bonferroni (dashed line) or permutation analysis (dotted line). (C) The candidate marker is associated with a significant 1.4-fold increase in susceptibility to the HIV.GFP. (D) rs2572886A is associated with a significant 1.6-fold increase in susceptibility of CD4^+^ T cells from healthy blood donors to replicating HIV-1, differences between the alleles remain significant (*p* = 0.0331) after removing the outliers.

To identify genomic loci that contribute to the variation in cellular permissiveness to HIV.GFP, we performed a quantitative genome-wide linkage analysis using a panel of 2,600 SNP markers with an effective resolution of 3.9 cM. Calculations were performed using the variance components analysis option from Merlin [21]. A region on HSA8q24 (marker rs1398296) showed the highest multipoint linkage score (logarithm of the odds [LOD] = 2.89, *p* = 1.3 × 10^−04^; Figure 2A). To determine the significance of this finding, we performed 500 simulations in which genotypes were randomized but the phenotype was kept constant, so as to preserve the heritability of the trait, the marker density, and the missing data patterns [22]. The distribution of maximum LOD scores of the simulations revealed that the observed HIV.GFP linkage peak is significant on a genome-wide basis at the 95% significance level (Figure 2A).

In order to independently confirm and fine-map the linkage analysis result, we assayed LCLs from 56 unrelated CEPH individuals that have been genotyped at a high density in the frame of the HapMap project [23]. The association analysis was performed using 521 tag SNPs in a 3-Mb region centered on the initial linkage assignment. A single SNP, rs2572886G>A, was strongly associated with HIV.GFP permissiveness (*p* = 1.8 × 10^−5^), and statistical significance was maintained after Bonferroni correction for multiple testing and permutation analysis (*n* = 10,000) (Figure 2B). Allele A of marker rs2572886 is associated with an average 1.4-fold increase in susceptibility to the HIV.GFP in LCLs from unrelated individuals, *p* = 0.001 (Figure 2C). Similar steps were taken for the secondary study phenotypes unrelated to lentiviral cellular susceptibility, which led to the precise identification (by linkage and followed by association) of a region involved in *cis*-regulation of *CD39* expression (Figure S2). In contrast, no locus was identified affecting LMP1 expression, suggesting a more complex control of this trait by multiple genes.

Because observations were all made on B cells, we next assessed the potential role of rs2572886 as a susceptibility factor for HIV-1 infection in CD4^+^ T cells. We genotyped the SNP in a collection of purified CD4^+^ T cells obtained from 128 Caucasian healthy blood donors. CD4^+^ T cells were infected with a replicating HIV-1, and permissiveness was assessed by p24 antigen production [3]. A significant association was again obtained for SNP rs2572886 and cellular susceptibility to HIV-1 on this independent sample (*p* = 0.019) using a biological system that more closely resembles the in vivo situation. Consistent with the results of transduction of B cells with a HIV-1–based vector, in CD4^+^ T cells, the allele A of marker rs2572886 is associated with a 1.6-fold increase in susceptibility to infectious HIV-1 virus than CD4^+^ T cells of noncarriers, as assessed in a 7-d replication kinetics analysis (Figure 2D). The size effect associated with rs2572886 in vitro is comparable to that identified for other genetic variants influencing the HIV life cycle [3].

**Figure 3:**
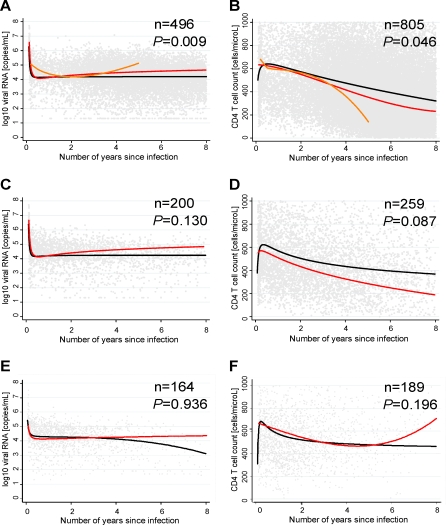
Assessment of rs2572886A in an HIV-1–Infected Human Cohort in the Absence of Therapy (A) Pattern of viral load in the full study population; each gray point represents a single viral load determination; the red (rs2572886A heterozygous), black line (individuals with the common allele), and orange lines (minor allele variant homozygous) represent the trend trajectories. (B) Pattern of CD4^+^ T cell depletion in the full study population; each gray point represents a single CD4^+^ T cell determination. (C) Pattern of viral load in infected individuals with known date of infection (incident sub-cohort). (D) Pattern of CD4^+^ T cell depletion in the incident sub-cohort. (E) Pattern of viral load in the validation incident cohort. (F) Pattern of CD4^+^ T cell depletion in the validation incident cohort.

Since the previous results were obtained from in vitro assays, we set out to assess the potential association of rs2572886 with disease progression in HIV-1 infected individuals. The rs2572886 SNP has a minor allele frequency of 7% in Caucasians (Utah CEPH individuals), 19% in West Africans (Yoruba Hapmap sample), and 23% in Asians (Han Chinese and Japanese, HapMap sample). We genotyped 805 individuals recruited in the frame of the genetic project of the Swiss HIV Cohort Study (http://www.shcs.ch) who provided informed consent. These patients contributed consecutive CD4^+^ T cell data (*n* = 4,999 measurements) and viremia (*n* = 1,926 measurements) over an average follow-up period of 7 y in the absence of anti-retroviral drug treatment. The rs2572886A allele was associated with greater viral load, and faster progression of immunosuppression, as defined by the slope of CD4^+^ T cells depletion over time (Figure 3A and 3B). Individuals homozygous for the minor allele variant (*n* = 7) exhibited, as a group, a faster disease progression, but no conclusions can be made with such a small sample size. The same trends were also present in the incident cohort of 259 individuals identified within a 1-y interval of seroconversion (Figure 3C and 3D), although the limited numbers precluded significant association. These results are consistent with the in vitro data, since the A allele, which was associated with higher susceptibility of infection in the cellular systems, was associated with greater viral load and faster progression in vivo.

As an additional validation step, we genotyped a second independent cohort including 189 individuals with a precise date of seroconversion (Figure 3E and 3F), which was collected in the context of a whole-genome association analysis [6]. No association was detected in this cohort; however, the power to detect association in a sample of this size is estimated to be around 25%. These patients were recruited by eight different cohorts, while the original results were established using Swiss HIV cohort data. When pooling the discovery incident subcohort and the validation cohort, the association of rs2572886 on both the CD4 and viral load did not reach significance despite the increased number of samples to 448. However, the combined sample is far from homogeneous. Thus this association should be considered as suggestive at this point..

The association results should be discussed in the frame of the recently published genome-wide association analysis of host determinants of viral setpoint [6]. First, the marker identified in the current study, rs2572886, is neither present nor tagged by the Illumina HumanHap550 BeadChip used in the paper by Fellay et al. [6]. In addition, the design and premises of the genome-wide association and those of the genome scan reported herein are different: (i) the manuscript by Fellay et al. led to the identification of acquired/innate immunity loci (in major histocompatibility complex [MHC]) that cannot be captured in a cellular assay that investigates the viral life cycle; (ii) the study design in the paper by Fellay was powered to detect only very strong and sufficiently common genetic determinants; (iii) the standardized infection conditions and the study endpoint (expression of a reporter) that were used in the current study are very different from the conditions encountered in a population of HIV-infected individuals. It is through the nature of these profound study design differences that we aimed at generating complementary information to that provided by the genome-wide association analyses**.**

To provide a reference parameter that would allow comparison with genetic variants identified in other studies, we estimated the proportion of variation explained by rs2572886 and compared it to the contribution of CCR5 Δ32 in the same study population. Including CCR5 Δ32 into the model increased the proportion of variation explained by 1.9% for the CD4 cell count and 0.4% for the viral load. For rs2572886A, the estimates were 0.8% and 1%, respectively. For comparison, age at infection contributed to an increase in the proportion of variation explained of 1.4% for the CD4 cell count and 0.05% for the viral load, whereas the increase was 3% and 3.1%, respectively, for gender. These values are comparable to estimates indicated in the literature for various genetic variants influencing HIV pathogenesis [24], although direct comparisons are difficult due to different study designs. In contrast, the proportion of variation explained by rs2572886 or CCR5 Δ32 is considerable smaller than that for the genetic determinants reported in the study by Fellay et al., reflecting the fact that this genome-wide association study was powered to detect strong genetic effects. Thus, *HCP5* and *HLA-C* variants explained 9.6% and 6.5% of the total variation in viral load, respectively, and an SNP near the *RNF39* and *ZNRD1* genes explained 5.8% of the total variation in disease progression [6].

The rs2572886 SNP is located in a nonconserved intergenic region on the telomeric end of Chromosome 8q (Figure S4). It is flanked on both sides by genes of the *LY6/uPAR* family (Figure S3). The *LY6* genes are characterized by conserved cysteine-rich domains with specific disulfide bonding patterns but with little homology (20%–30% amino acid conservation among family members); members are either glycosyl-phosphatidyl-inositol (GPI)–anchored cell-surface receptors or secreted cytotoxins. Eight genes are located at 8q24.3: *LY6K*, *SLURP1*, *LYPD2*, *LYNX1*, *LY6D*, *GML*, *LY6E*, and *LY6H.* The functions of the encoded proteins are diverse but not well understood [25]. None has been associated with HIV-1 in the past, although the *LY6H* gene was reported to be up-regulated upon HIV infection [26]. A related protein of the LY6/uPAR family, the urokinase-type plasminogen activator receptor, coded by a gene in Chromosome 19, has been reported up-regulated in HIV-infected individuals, and proposed to participate in the innate immunity to HIV-1 through an interferon (IFN)-like mechanism [27,28].

The SNP rs2572886 is located in a recombination hot spot between two linkage disequilibrium blocks. We resequenced the surrounding region (∼ 13 kb) in 30 chromosomes to identify additional SNPs in linkage disequilibrium with rs2572886 that might point toward a biological function. Although two closely positioned SNPs—rs12546765 and rs12546801—were associated with rs2572886 in this limited resequencing dataset, they are not found in linkage disequilibrium in HapMap (pairwise *r* ^2^ = 0.03). The region (∼1 kb) where rs2572886 is located is only present once in the human genome. We downloaded the homologous region of chimpanzee and Rhesus macaque and sequenced it in seven additional primates (bonobo, gorilla, orang-utan, nomascus gibbon, siamang gibbon, baboon, and African green monkey). rs2572886G>A was particularly variable among primates, with “G” representing the ancestral nucleotide in Old World monkeys, “A” the ancestral residue in hominoids, and “T” in gibbons.

**Figure 4:**
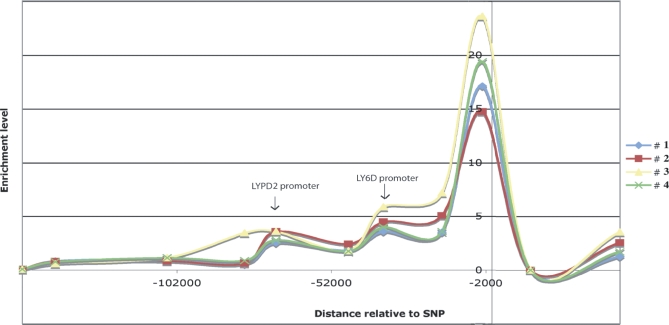
Long-Range Interactions between rs2572886 and LY6/uPAR Gene Promoters Curves show results of quantitative chromatin conformation capture (3C) for four independent CD4^+^ cell lines. The *y*-axis corresponds to interaction enrichment relative to background (furthest point) and corrected according randomly ligated BAC. The *x*-axis shows relative distance to rs2572886.

To identify candidate genes that could be functionally related to the rs2572886 SNP, we performed quantitative 3C (chromatin conformation capture) [29], with the goal of detecting potential chromatin interactions between the SNP region and neighboring genes. We tested 11 regions by Taqman real-time PCR, spanning a distance of 190 kb surrounding the SNP. We focused primarily on the upstream areas (promoters) of genes in the locus. Results from cross-linked cells were compared to randomly ligated BAC DNA from the same region to correct for interassay differences and potential ligation biases. As expected, we observed a high level of enrichment with a region located 3.1 kb from the SNP (positive control) due to random chromatin interactions that have been reported to occur between regions separated by less than 5 kb [30]. The trend for enrichment rapidly decreased with increasing distance. Interestingly, we detected higher than background peaks of enrichment on the upstream areas of two genes—*LY6D* and *LYPD2* (Figure 4)—suggesting that these are good candidates for functional interaction with the associated SNP. There are no apparent interactions between the SNP and the nearby GML promoter, despite its relative proximity (12 kb) in comparison to the *LY6D* (35 kb) and *LYPD2* (70 kb) genes.

We prioritized the following proteins for additional biological assessment: LY6D and LYPD2 on the basis of chromatin conformation capture analysis, SLURP1 based on its unique status of secreted protein, and GML because of the proximity to the genetic marker. First, we overexpressed each of the four proteins from several vector backgrounds in 293T and HeLa cells to assess whether this would influence transduction by HIV.GFP. No significant changes in cellular infectivity were detected upon overexpression in these cell lines (Table S1).

In general, all eight genes of the *LY6/uPAR* family show detectable, but very low levels of expression as assessed by quantitative RT-PCR (unpublished); this precludes gene expression variation analysis to determine whether the genotype at rs2572886 correlates with expression levels of nearby genes. *LY6D* and *LYPD2*, which showed relatively higher expression levels, were silenced in HeLa cells by small interfering RNA (siRNA). Silencing with three different siRNA was successful for *LY6D* and suboptimal for *LY6PD2*. After transduction with HIV.GFP, we observed minor modifications in rates of cellular infection (Table S1). These findings are interesting, but given the cell type used and the harsh treatment of the cells, they are not conclusive enough to make a functional link for these genes. Overall, the biological basis for a role the LY6/uPAR family of proteins in HIV-1 cellular susceptibility remains elusive after this first line of biological screening. Additional analyses will be required to convincingly demonstrate a role for these proteins in the HIV life cycle.

In summary, by using a multi-step procedure involving a whole-genome linkage scan followed by association studies, we identified a locus on HSA8q24 that influences cellular susceptibility to HIV-1, and possibly progression of HIV-1 infection in vivo. Although the initial findings were based on transduction of transformed B lymphoblastoid cells with a HIV-1–based vector, subsequent experiments first on primary CD4^+^ T cells infected with replicating HIV, and second on a cohort of untreated HIV patients, supported the initial observations of association. In addition, although quantitative 3C data suggest a possible participation of genes of the LY6/uPAR family, further work is required to decipher the biological mechanism underlying this association.

## Materials and Methods

### Plasmids.

Members of the LY6/uPAR family—*LY6D* (GenBank NM_003695), *LYPD2* (NM_205545), *GML* (NM_002066, SC303114; OriGene), *Lynx1c* (NM_177457), *Slurp1* (NM_020427), and *Slurp2* (NM_177458)—were amplified by RT-PCR from RNA extracted from human cell lines (HeLa or 293T), and cloned into pCI-neo. *LY6D* and *LYPD2* were tagged C-terminally with an HA tag.

### Cell culture.

293T cells were cultivated in Dulbecco's modified Eagle Medium (DMEM; Invitrogen) supplemented with 10% heat-inactivated fetal bovine serum (FBS) and 50 μg/ml gentamycin. 293T cells (3 × 10^6^ cells) were cotransfected with 20 μg total of DNA (empty pCI vector + increasing amounts of pCI vector containing the gene of interest) using the calcium phosphate technique. Twelve hours post-transfection, cells were washed and 300,000 cells were seeded in six-well plates and incubated further for 36 h to allow the expression of the gene of interest before HIV-based vector infection.

### CEPH cell lines.

EBV-transformed B cell lines were obtained from the CEPH collection through the Coriell Institute for Medical Research (http://locus.umdnj.edu/nigms/ceph/ceph.html). Cells were cultivated in RPMI 1640/Glutamax-I medium (Invitrogen) supplemented with 15% fetal calf serum (FCS, Inotech). They were maintained by replacing half medium twice a week. Pedigrees studied were numbers 102, 884, 1328, 1331, 1332, 1333, 1334, 1340, 1341, 1345, 1346, 1347, 1362, 1408, and 13292.

### CD4^+^ T cell isolation and B cell immortalization.

Cells from 11 white healthy blood donors were used to isolate CD4^+^ T cells by using anti-CD4 magnetic beads (Miltenyi Biotech). Cells were cultured in RPMI1640/Glutamax-I medium supplemented with 20% FCS, 20 U/ml human interleukin-2 (IL-2, Roche) and 50 μg/ml gentamicin (Invitrogen) following stimulation with phytohemagglutinin (PHA) at 2 mg/ml for 2 d [31]. The CD4-negative cell fraction was exposed to EBV containing supernatant from a B95–8 cell line according to current protocols [32].

### HIV-based vector production.

To produce HIV-based vector particles (HIV.GFP), 293T cells (3 × 10^6^ cells) were cotransfected with four plasmids using the calcium phosphate method. Plasmids encoded the VSV-G pantropic envelope (pMD.G), the Gag and Pol proteins (pCMVΔR8.92), Rev (pRSV-Rev), and the fourth plasmid encoded the HIV vector segment carrying GFP as the reporter transgene under the control of the CMV promoter (pWPTS-GFP) (kind gifts from D.Trono, EPFL, Lausanne, Switzerland; see http://rd.plos.org/pbio.0060032.1 for vector details). Forty-eight hours after transfection, the supernatant was collected, centrifuged to pellet cellular debris and filtered through 0.45-μm filters. Viral particles were concentrated by centrifugation through a 100-kDa cut-off membrane (Centricon Plus-70; Millipore AG).

### HIV-based vector transduction (single-round infectivity assay).

Transduction of CEPH cells and of the B cells of healthy blood donors (0.5 × 10^4^ cells in 96 wells) was performed by spinoculation with HIV-based particles for 3 h at 1500*g* and 22 °C. After 72 h, cells were harvested and expression of GFP protein was monitored by fluorescence activated cell sorting (FACS) analysis with mock transduced cells as control. This was performed twice in triplicate at 1-wk intervals. Similarly, transfected 293T cells (in six-well plates) were infected with HIV-based particles (25 ng p24 equivalent) in the presence of 10 μg/ml DEAE-dextran in 1 ml D-10 for 5 h. Culture medium was replaced and cells were incubated for 24 h before FACS analysis of GFP expression. Typically, 20%–30% of GFP^+^ cells were obtained for controls.

### HIV infection.

Healthy blood donors' CD4^+^ T cells (10^6^ cells) were infected with NL4-3BaL virus (1,000 pg of p24 antigen) in a 1-ml final volume for 2 h at 37 °C in 5% CO_2_. Cells were washed and cultured for 7 d. Virus-containing supernatant was harvested, and p24 antigen production was monitored by an enzyme-linked immunosorbent assay (ELISA) (Abbott).

### Phenotyping.

For cell surface molecule staining, 10^5^ CEPH cells were washed, resuspended in PBS/0.5% BSA (Sigma) and incubated with primary monoclonal antibodies (mAbs) or isotypes for 15 min at room temperature (RT). Primary mAbs were : anti-CD11a (Dako, MHM24), -CD19 (Dako, HD37), -CD21 (Dako, 1F8), -CD23 (Dako, MHM6), -CD39 (Serotec, A1), -CD54 (Dako, 6.5B5), and the negative control was mouse IgG1FITC (Dako, X0927). All mAbs were used at 1/50, except CD19 used at 1/20. For intracellular staining, CEPH cells were washed and resuspended in cytofix/cytoperm solution (Becton Dickinson) for 20 min at 4 °C. After two washes with permwash, cells were resuspended in permwash with primary anti-LMP1 (1/50, S12, a gift from S. Rothenberger) or negative control antibody mouse IgG2a (1/50, Dako), for 15 min, at RT. After washes, cells were incubated in permwash with secondary anti-mouse PE (1/30, Dako). After wash, cells were fixed (CellFix, Becton Dickinson) and analyzed using a FACSCalibur system for 10,000 events. Positive events were defined as a fluorescence level superior to that of isotypic control. Determination of EBV copy number was carried out by real-time PCR by using specific probes as described [33].

### Heritability, linkage, simulations, and associations studies.

Heritability calculations (*h* ^2^) were performed using the “polygenicscreen” command from the SOLAR software [34]. SNP genotyping data, consisting of 2,688 autosomal SNPs were downloaded from the SNP Consortium database (http://snpdata.cshl.edu/population_studies/linkage_maps/) [35]. Multipoint linkage with the SNP map was performed using Merlin [36] with the –VC option, after Mendelian inconsistencies (PEDCHECK) [37] and unlikely genotypes (PEDWIPE) [38] were removed. To calculate the empirical significance of the linkage results, we performed 500 simulations for each quantitative trait using the –simulate command from Merlin with different seed numbers. We extracted the highest result from each simulation to build significance distributions. All simulations were performed using a cluster of 32 HP/Intel Itanium 2 based servers at the Vital-IT Center (http://www.vital-it.ch/). Association analysis of quantitative phenotypes (% of GFP-positive cells and / mean fluorescence intensity (MFI) of CD39), and corrections for multiple testing were performed using the PLINK software (http://pngu.mgh.harvard.edu/~purcell/plink/anal.shtml). Genotypes were downloaded from the HapMap project URL (http://www.hapmap.org/cgi-perl/gbrowse/gbrowse/hapmap/), HapMap public release number 19.

### Cohort analysis.

Data from both incident (patients identified during primary infection or who have had a negative and positive test for HIV infection within a narrow time interval, 1 y in this study, in which case the date of infection is estimated as the mid-point), as well as data from prevalent cases (i.e., individuals already HIV-seropositive by the time they entered the study, unknown date of infection) were analysed longitudinally by modeling the CD4 T cell count and HIV-1 RNA marker's trajectories over time for the different genotype groups. The analysis was conducted using population-averaged marginal modeling [39], because the focus of the study was to investigate the effect of specific genetic factors on disease progression at the population level. In a marginal model, the mean regression function is modeled independently from the variance–covariances matrix. We used fractional polynomials to assess the best-fitting functional form. The viral load (log scale) and the CD4 (square root scale) trajectories post seroconversion were linear and appeared stationary. Therefore, linear functions of time, along with interactions with polymorphisms and covariables (age at infection and gender) were considered. The impact of genotype on slope and intercept was assessed using Wald test, and the proportion of explained variation was assessed [40]. A multivariate distribution was fitted to the data by score-like methods (generalized estimating equations) [41]. The correlation structure was assumed to be well represented by an autoregressive process of order 1. To limit the impact of frailty selection, only the data for the first eight years since seroconversion were considered. The analysis was repeated considering, in turn, only the incident, prevalent, and both cohorts. Subanalyses were also performed considering the Caucasian group only. For the prevalent cases, an estimate of the unknown date of infection was obtained using the markers data and defining for each patient an infection window based on his or her last negative and first positive available HIV tests. The date of infection was then imputed using a methodology that extends published methods [42,43] to accommodate multiple marker measurements per individual (P. Taffe and M. May, unpulished data, and [44]. A second incident cohort, recruiting individuals from various European countries, was used to validate results obtained from the analysis of the incident cohort recruited within the Swiss HIV Cohort study. Statistical analyses were conducted using SAS version 9.1 for Windows, as well as STATA 9.2 .

### Re-sequencing in human and nonhuman primates.

The region (∼1 kb) around the candidate marker was resequenced by using forward primer SG2000 (5′-AGTTCATACCCCTTTGCCAGGTTG) and reverse primer SG2001 (5′-GAAGCCTTACCTGCTTCCTGCC), and forward primer SG1829 (5′-TTCCCTGAGCTTGCAGGACTC) and reverse primer SG1853 (5′-CTCTACACACCTACCTTGCTGGGA) to generate overlapping PCR products.

Sequences have been submitted to GeneBank for bonobo (EU340888, Pan paniscus), gorilla (EU340889, Gorilla gorilla), bornean orang-utan (EU340890 , Pongo pygmaeus), nomascus (EU340891, Hylobates leucogenys), siamang (EU340892, Hylobates syndactylus), baboon (EU340893 Papio hamadryas), and African green monkey (EU340894, *Cercopithecus [chlorocebus] aethiops*).

### Quantitative chromatin conformation capture.

Approximately 10^7^ stimulated expanded primary CD4^+^ T cells [31] were crosslinked in their media for 10 min at RT with 1% formaldehyde (v/v). Crosslinking was quenched with 125 mM glycine prior to two successive washes with 1xPBS. Pelleted cells were resuspended into 5-ml ice-cold lysis buffer (10mM Tris HCl, pH 8.0, 10 mM NaCl, 0.2% (v/v) NP-40) complemented with protease inhibitors (Complete, Roche) and 0.5 mM PMSF. Lysis of the cells was allowed to proceed for 10 min on ice with mild shaking. Nuclei were recovered by centrifugation (600*g* for 5 min at 4 °C) and resuspended into 500-μl 1.2xDpnII restriction buffer (NEB). Nuclei were lysed with 0.3% (v/v) SDS for 60 min at 37 °C. SDS was sequestered with 2% (v/v) Triton X-100 for another 60 min at 37 °C. Chromatin was subsequently restricted overnight at 37 °C with 500 U DpnII (NEB) in a final reaction volume of 600 μl. After heat inactivation of the restriction enzyme (10 min at 65 °C), chromatin was dialyzed (Slide-A-Lyzer, Pierce) for 1 h against 1.5 l of water at RT and transferred into 7 ml ligation reaction mix (50 mM Tris HCl, pH 8.0, 10 mM MgCl2, 0.5mg/ml BSA, 10mM β-mercapto-ethanol, 0.5 mM ATP and 400 U T4 DNA ligase (NEB). The ligation reaction was performed for 4 h at 16 °C followed by another 30 min at RT. Crosslinking was heat-reversed and proteins were degraded (300 μg proteinase K) overnight at 65 °C in a hybridization oven. DNA was purified by phenol/chloroform/isoamyl alcohol [25:24:1(v/v)] extraction, precipitated with isopropanol, and washed with ethanol 70%. DNA was subsequently resuspended with 200 μl 1xTE pH 8.0 and treated with 50 μg RNaseA for 30 min at 37 °C. Finally, DNA was extracted with 1 volume phenol/chloroform/isoamyl alcohol [25:24:1(v/v)], ethanol precipitated, and resuspended into 100 μl 1xTE pH 8.0. Cross-linking was independently performed on four CD4^+^ lines derived from different individuals, two of whom (2 and 4) were heterozygous for rs2572886.

For quantitative Taqman PCR, we designed 11 assays comprising the PCR primers and a dual-labeled probe sitting at the predicted DpnII junction between the target and bait regions (primer and probe sequences are available upon request). Reactions were set up using a Biomek 2000 robot (Beckman), in a 10-μl volume in 384-well plates. Three replicates per assay per sample were performed. PCRs were run in an ABI 7900 Sequence Detection System (Applied Biosystems) with the following conditions: 50 °C for 2 min, 95 °C for 10min, and 50 cycles of 95 °C 15 s/60 °C for 1 min. Each reaction contained 300 nM of each primer and 250 nM of probe.

For the 3C samples, approximately 200 ng of DNA was used per well, and for the BAC (digested – randomly ligated) samples, 10 ng of DNA was used. Normalization for each assay was performed using the values obtained from BAC experiment (all assays are expected to give the same result, given that the naked BAC was fully digested and re-ligated, and all ligation combinations are expected to be present equimolarly). Enrichment was calculated with respect to the most centromeric probes, which showed very low levels of interaction.

***Note in Press:*** Recently, Brass et al [45] identified through a siRNA screen over 250 HIV-dependency factors. Among these there were three members of the LY6/uPAR family (GML, LY6D, and LYPD4). This new evidence provides independent support for a biological role of the LY6/uPAR family in HIV-1 pathogenesis.

## Supporting Information

Figure S1 Validation of the CEPH Model to Study HIV-1 Susceptibility CD4^+^ T cells and B cells from same healthy blood donors were transduced with the HIV.GFP. Linear regression of one representative experiment for the percentage of GFP-positive cells is shown. Each point represents the mean of triplicate values.
(41 KB PPT)

Figure S2 Expression and Heritability of HIV-1 Susceptibility Trait in CEPH Families (A) Heritability estimate (*h* ^2^) for eight study traits (percent of positive cells and mean fluorescence intensity, MFI), and for the EBV copy number (** *p* < 0.001, *** *p* < 0.0001).
(B) Representation of interindividual (black dots), and interfamily (red bar = median value for each family) differences in percentage of GFP-positive cells after transduction with a HIV.GFP of 198 lymphoblastoid B cell lines from 15 CEPH pedigrees.
(68 KB PPT)

Figure S3 Genome Scan and Fine Mapping of CD39 Expression (MFI Trait) (A) Results of linkage analysis with 15 families. A single locus on HSA10q23 (rs766083) is significant after correction by permutation analysis (*n* = 500).
(B) Results of association analysis in 56 independent individuals using SNPs from the HapMap project. All SNPs in a 700 kb region surrounding the CD39 gene were used. rs numbers next to peaks indicate significant SNPs after correction for multiple testing.
(94 KB PPT)

Figure S4 Chromosomal Localization of rs2572886 The SNP is localized in an intergenic region containing genes belonging to the *LY6/uPAR* family. The figure shows images generated through the University of California at Santa Cruz genome browser and Haploview software.
(168 KB PPT)

Table S1 Infectivity of HIV.GFP of Cells Transfected with or Silenced for the Gene of Interest from the *LY6/uPAR* Family Values reflect relative infectivity to that of the mock control. None of the differences are statistically significant.
(26 KB DOC)

## Abbreviations

CEPH: Centre d'Etude du Polymorphisme Humain
GFP: green fluorescent protein
GPI: glycosyl-phosphatidyl-inositol
LCL: lymphoblastoid B cell lines
LY6: lymphocyte antigen 6
SNP: single nucleotide polymorphism
