# Supplementary material for: In Vitro Whole-Genome Analysis Identifies a Susceptibility Locus for HIV-1
Source: PLoS Biol. 2008 Feb 19;6(2):e32. doi: 10.1371/journal.pbio.0060032 (PMC2245987; doi:10.1371/journal.pbio.0060032)
Supplement: Supplementary file 1 [file pbio.0060032.st001.doc]

**Table S1** : Infectivity of HIV.GFP of cells transfected with or silenced for the gene of interest from the LY6/uPAR family. Values reflect relative infectivity to that of the mock control. None of the differences are statistically significant.

|  | Overexpression (293T) | | Overexpression (HeLa) | | Silencing (HeLa) | |
| --- | --- | --- | --- | --- | --- | --- |
| Gene | Mode of  assessment of expression | Relative Infectivity (MFI) | Mode of assessment of expression | Relative Infectivity (MFI) | % knockdown (Taqman) | Relative Infectivity (MFI) |
| Slurp1 | Real-time PCR | 98.03 (106.18) | ND | ND | ND** | ND |
| LY6D | WB* | 99.56 (109.78) | WB | 88.36 (71.45) | > 80% | 105.90 (122.10) |
| LYPD2 | WB | 98.78 (108.19) | WB | 79.73 (79.74) | 34% | 93.73 (57.99) |
| GML | ND | 83.35 (110.05) | WB | 108.10 (117.19) | ND** | ND |

*Poor expression

**siRNA not done because of low levels of natural, endogenous mRNA expression.

MFI, mean fluorescent intensity; WB, Western blot; ND, not done
